# Do Working Men Think?

**Published:** 1890-01-18

**Authors:** 


### The Hospital

A Weekly Journal of

Science, Medicine, Nursing, and Philanthropy.

*For Hospitals, Asylums, Medical Practitioners, Students, Nurses, Families, Parishes, Congregations, and the Charitable Public.*

Vol. VII. No. 173.] [January 18, 1890.

### Do Working Men Think?

The question as to whether the average British working-man is able to think out a subject dispassionately and without prejudice, is induced by the consideration of certain facts which transpired at Exeter a week or two ago. The committee of the Hospital Saturday Fund of that West of England city met for business, and one of the delegates expressed himself in exceedingly plain terms as to the rights and claims of working-men on the benefits of voluntary medical charities. He said, for example, that working-men were "entitled" to the benefits of institutions which they "supported." He asked "how could the committee go to fellow working-men for support to institutions the by-laws of which debarred them their benefits?" He proceeded to say that "the Eye Infirmary, or any other institution that was open to receive subscriptions from working-men, should make its rules to suit the working-men." He then went on to propose that "the committee should withhold all assistance from the infirmary to show the disgust of the working classes at the treatment they had received." The occasion of this plain speaking seems to have been the rule of the hospital, which provides that a man whose wages amount to twenty-five shillings per week, or more, is not a suitable person for charitable medical relief.

This working-man delegate discussed a question of much practical importance to philanthropists and working-men with great plainness of speech. He and his fellow-workmen will perhaps permit us to speak with equal plainness. The speaker used the words "entitled" and "supported," and laid down the general principle that working-men are "entitled" to the benefits of hospitals because they "support" them. But if this ground is to be taken by working-men, they should at least understand the exact position in which they elect to place themselves. They demand a *quid pro quo* when they subscribe. That is to say, they give something to hospitals for which they expect to get a return. All this sounds extremely fair and proper, and no doubt quite commends itself to the plain common-sense of the plain working-man. But is it, or is it not, one of the most elementary principles of everyday business that when a man gives something for which he expects a return there shall be a certain proportion between the value of the gift and that of the return? No man who gives five pounds for the purpose of securing a return expects to get back fifty, or a hundred, or a thousand, or ten thousand. If he receives exactly his money's worth he considers himself quite justly treated.

Now let these principles be applied to the case of the Exeter Eye Infirmary and the Exeter working-men. The men gave last year to the infirmary the sum of £37 12s. 7d. If their own principles are accepted, they were "entitled" to the value of £37 12s. 7d. in special medical and surgical treatment; that and no more. Did the working classes receive the value of £37 12s. 7d. in special medical and surgical treatment during the past year at the Exeter Eye Infirmary? They received, probably, not ten nor twenty, but fifty or a hundred times the value of that amount; or, in other words, fifty or a hundred times more than they were on any reasonable ground whatever "entitled" to claim.

But the logical champion of the Exeter working-men put forward claims more startling even than these. "If," said he, "the Eye Infirmary, or any other institution, was open to receive subscriptions from working-men, it should make its rules to suit the working-men." How very obvious and conclusive that would sound in the ears of a working class audience! But let us consider another fact bearing upon this question. Sir Andrew Clark is the president of the Royal College of Physicians, and Mr. Jonathan Hutchinson the president of the Royal College of Surgeons. Both those distinguished men are open to receive fees at the hands of their private patients, exactly as the Exeter Eye Infirmary is open to receive subscriptions from the Exeter working-men; but with this difference, that whilst the fee paid by each patient to either of those gentlemen may range from two to two thousand guineas, the subscriptions given by the Exeter working-men to the Eye Infirmary would not amount to more than a fraction of a farthing for each person treated. Now Sir Andrew Clark and Mr. Jonathan Hutchinson both have rules according to which they conduct their practice, exactly as a hospital has rules for the conduct of its work. But who makes the rules for those eminent practitioners, and whose convenience are those rules framed to accommodate? Sir Andrew Clark and Mr. Hutchinson both make their own rules for their own convenience; and every man and woman, however rich or great, who consults either of those practitioners must do so at the times and under the regulations laid down by the practitioners themselves. What have working-men to say to this, when they compare it with the monstrous and preposterous claims they put forward as the equivalents of the utterly insignificant sums they subscribe as a body to the medical charities? These facts, and the question they suggest, demand a rational consideration at the hands of every fair-minded workman, both in Exeter and elsewhere.

It is certain that hospital managers, as a class, sympathise with the disadvantages on the one hand, and the laudable aspirations on the other, of working-men. They desire to make such regulations at hospitals as will confer upon patients the maximum of benefit with the minimum of inconvenience. Not only so, but they regard with favour, and actively promote, the introduction of working-men to the councils, committees, and other governing bodies of medical charities. These things they do because they think the best interests, both of the working classes and of the charities, will be thereby served. The example of the managers of the Coventry Infirmary is a striking case in point. Working-men have thus, without difficulty, secured a larger share of representation and influence in the management of hospitals than the amount of their contributions at all entitles them to. It is just possible that they may in their own minds be applying to hospitals the principle so popular with a certain class of politicians of "one man one vote." But they must remember that a state is one thing and a voluntary association another. An ordinary hospital is a voluntary association, of which, by constitution, the subscribers of a certain minimum sum annually are the constituents and ultimate court of appeal. Just as the private individual who subscribes a smaller amount than the qualifying subscription never thinks of claiming any right to vote or to demand medical treatment, so neither can any body of men claim any sort of rights individually, whatever be the amount of their aggregate subscriptions, so long as each person fails to contribute such a sum as will qualify him to become a constituent member of the association. Bodies of working-men stand in exactly the same relation to hospitals as churches and other public bodies. A church or firm subscribing to a hospital is entitled to such a measure of representation and to such benefits as are strictly proportioned to the amount of its contributions. Bodies of working-men contributing to hospitals are entitled, and justly, to exactly the same measure of proportionate representation and advantage, and no more. That is surely as fair a state of things as can well be imagined. But to suppose that a working-man delegate who represents, say, forty pounds of subscriptions, on the management of a hospital, the annual expenditure of which is fifty or sixty times forty pounds, is entitled to construct or alter the rules of the hospital so as to suit the sole convenience of working-men, is one of those propositions which for sheer unreasonableness and absurdity can only be matched in such books as "Gulliver's Travels" or "The Pathetic Romance of a Scare-crow."

It is plain that working-men are very much at sea with regard to hospitals and their management, and also with regard to their own rights and privileges therein. The truth is that they have no "rights" at all, but only "privileges." They can acquire rights by paying for them; and they can destroy privileges by claiming them as rights. It is to be feared that they allow themselves to be led astray by mere irresponsible talkers of their own class. Many of those talkers either do not understand the subject, or take it for granted that their fellow-workmen do not. The great point to be borne in mind is that practically all hospitals are voluntary charities, and not State institutions. Nothing is easier than for the subscribers of any hospital to withhold their subscriptions, and then the work of the hospital must fall to the ground. The question is—Will the working-men because they subscribe, say, one pound of every hundred to the cost of hospital maintenance, run the risk of losing the other ninety-nine pounds by claiming rights which have no existence, instead of taking gratefully the privileges and advantages which the voluntary supporters of hospitals have hitherto so generously conferred upon them? Working-men and their friends will do well to consider the subject from the standpoint here taken. If they will approach it with reason they will be met by reason. If they behave like unthinking and irresponsible children, they must not be surprised if they find the timid birds of voluntary charity flying away beyond the reach of their foolish clamour.

